# Bioinspired Implementation and Assessment of a Remote-Controlled Robot

**DOI:** 10.1155/2019/8575607

**Published:** 2019-09-11

**Authors:** Yves Rybarczyk, Diogo Gil Carvalho

**Affiliations:** ^1^Intelligent & Interactive Systems Lab (SI2 Lab), Universidad de Las Américas, 170125 Quito, Ecuador; ^2^Dalarna University, 791-88 Falun, Sweden; ^3^Department of Electrical Engineering, CTS/UNINOVA, Nova University of Lisbon, 2829-516 Monte de Caparica, Portugal

## Abstract

Daily activities are characterized by an increasing interaction with smart machines that present a certain level of autonomy. However, the intelligence of such electronic devices is not always transparent for the end user. This study is aimed at assessing the quality of the remote control of a mobile robot whether the artefact exhibits a human-like behavior or not. The bioinspired behavior implemented in the robot is the well-described two-thirds power law. The performance of participants who teleoperate the semiautonomous vehicle implementing the biological law is compared to a manual and nonbiological mode of control. The results show that the time required to complete the path and the number of collisions with obstacles are significantly lower in the biological condition than in the two other conditions. Also, the highest percentage of occurrences of curvilinear or smooth trajectories are obtained when the steering is assisted by an integration of the power law in the robot's way of working. This advanced analysis of the performance based on the naturalness of the movement kinematics provides a refined evaluation of the quality of the Human-Machine Interaction (HMI). This finding is consistent with the hypothesis of a relationship between the power law and jerk minimization. In addition, the outcome of this study supports the theory of a CNS origin of the power law. The discussion addresses the implications of the anthropocentric approach to enhance the HMI.

## 1. Introduction

Industries face an increasing demand for collaborative robots that exhibit human-like behaviors. This trend is justified by the fact that it is easier for an operator to predict the actions of a robot that behaves more like a human being than like a machine [1]. A study that uses the experimental paradigm of Motor Interference (MI) shows that the motor performance of an individual can be influenced by the perception of the movements of a robot, if the machine replicates some characteristics of biological motion [2–4]. In particular, it seems that the movement velocity profile is sufficient to create this interference. This result suggests that a movement can be processed as biologic by the human brain, even if it is not produced by a living being, on the condition that the artefact motion simulates (even approximatively) certain biological kinematics [5]. Moreover, the physical aspect of the robot seems relevant. For instance, the human-robot interaction tends to be improved when the machine has a humanoid appearance [6]. This fact can be explained by an inconscient tendency of the human being to anthropomorphize the artefacts they interact with, in order to predict their behavior and increase their acceptance of the machine [7].

Besides the situation of interaction, the implementation of human-like behaviors in a robot's way of working also seems to benefit an operator that has to control a machine. This statement is particularly true in the context of teleoperation, which implies several limitations for a human operator. For instance, the sensorial information received by the teleoperator can be altered, for example, the field of view is reduced, not all the sensorial modalities are restituted (e.g., audition and proprioception), and the response of the system is delayed. Another aspect is the necessity to build or accommodate new motor schemes to be able to control the user interface of the device, which augments the mental workload. A promising approach to reduce the gap between the user and the telerobot is to implement human-like behaviors in a robot [8, 9]. For instance, Rybarczyk et al. [10, 11] have studied the effect of the implementation in a mobile robot of the human behavior of visuomotor anticipation over the locomotion, in which the direction of the robot pan-tilt camera is automatically oriented toward the tangent point of the inside curve of the path, as walkers/cyclists/drivers do [12–14]. The results show that the motor performances of the teleoperators are enhanced when they steer the bioinspired robot. A correlation between the replication of biological laws and the level of expertise is also observed in the case of the telemanipulation of robotic arms, such as in telesurgery [15].

Different strategies are used to implement human-like behaviors in a robot. A traditional approach applied in the industry is to create anthropomorphic collaborative robots (or cobots) that are trained to imitate biological motions, through machine learning algorithms [16]. In the case of the teleoperation, it seems that individuals feel also more comfortable to control an anthropomorphic robot arm in which the motion trajectory of the end effector is like a biological movement [17]. Jerk minimization is one of the principal human-like behaviors that has been implemented to model a natural trajectory planning [18, 19]. The minimum jerk is characterized by a bell-shaped velocity profile, in which the movement speed increases progressively, reaches a peak near the midpoint, and then deceases slowly. This absence of abrupt changes seems to support the execution of a smooth motion [20]. Another fundamental motor behavior is the relationship between the velocity and the curvature of the biological movements, which is known as the two-thirds power law [21, 22]. This law states that the angular velocity of the end effector is proportional to the two-third root of its curvature or, equivalently, that the instantaneous tangential velocity (*v_t_*) is proportional to the third root of the radius of curvature (*r_t_*), as described in equation (1). In other words, it means that the velocity of the movement decreases in the highly curved parts of the trajectory and increases when the trajectory becomes straighter. Implementing this model in a mobile robot tends to improve the raw performance when steering the vehicle [23].

(1) $$\begin{array}{c} v_{t}=k\;{r_{t}}^{-1/3}. \end{array}$$

Nevertheless, few studies are interested in considering refined features to gauge the quality of the Human-Machine Interaction (HMI). Instead of focusing only on the raw performance (e.g., completion time of the task and percentage occurrence of errors), these studies analyze the kinematics of the robot control [24–26]. To proceed with such an advanced assessment, the human behaviors are now used as criteria to estimate an appropriate interaction. For instance, minimum jerk, smoothness, and 2/3 power law can be applied as a reference to evaluate a suitable interaction between a human operator and an artefact [17, 27]. These three features are compared in a study that aimed at assessing the motor control of a robot arm to assist surgeons [15]. The results show that both smoothness and minimum jerk are significant measures of expertise levels. The end-effector trajectory evolves from sharp and jerky in novices to smooth in experts. Thus, the authors conclude that these two features are excellent criteria to evaluate motor skill in the conditions of human-robot interaction. Although the power law is also identified as a discriminant measure of expertise, registering such a biological law seems to depend on the characteristics of the artefact. For example, some studies have demonstrated that this law is replicated in situations of teleoperation [11] and use of prostheses [28].

Actually, there is a controversy regarding the origins and the violations of the 2/3 power law during the execution of the biological movements [22, 29–31]. On the one hand, some studies tend to demonstrate that the power law is a signature of the Central Nervous System (CNS) [32–34], because it seems to be independent of the dynamics of the limbs. This law is indeed observed in a wide variety of activities such as drawing [21], walking [35], and smooth pursuit eye [36]. On the other hand, different studies defend a biomechanical [30] or, even, an artefactual explanation [37, 38]. There are also contradictory results regarding the relationship between smoothness, minimum jerk, and power law. Some studies show evidences that these features are related to each other [32, 39], whereas others suggest the contrary [15, 29].

The present work attempts to tackle these different contradictory findings about the 2/3 power law by integrating this bioinspired kinematics in a remote-controlled mobile robot. An experiment is designed to compare the teleoperation of a robot with the 2/3 power law (biological condition) versus two modes of control that do not implement this human-like behavior (manual condition and nonbiological condition). In the biological mode, the engine speed is automatically servo controlled by the vehicle trajectory according to the power law equation. In the manual mode, the user has to control both the velocity and the direction of the mobile device. In the third condition, the vehicle speed is also automatic, but the calculation of the relationship between geometry and kinematics violates the biological motion. This last condition is used as a control to make sure that the potential difference of performance between the two main conditions (biologic vs. manual) is not caused by a dissimilar complexity of the task (i.e., number of parameters that must be controlled by the participants). We posit the hypothesis that semiautonomous driving, in which the velocity is automatically set according to the power law principles (biological mode), should promote a significantly faster, safer, and more natural steering than the nonassisted (manual mode) and nonbiologic (artificial mode) control. The quality of the interaction is assessed from both the raw performance (completion time and number of collisions) and refined parameters based on the smoothness of the trajectories.

The remainder of the manuscript is organized into three main sections. First, the implementation of the teleoperation system is described. The experimental protocol and conditions (manual vs. nonbiologic vs. biologic) are also explained in detail. Second, the results of the performance for each condition are presented, analyzed, and discussed. Finally, the outcomes are interpreted, in order to draw some conclusions and perspectives regarding the application of the anthropocentric approach in the human-robot interaction, as well as the origins of the power law and its relationship with jerk minimization.

## 2. Material and Methods

### 2.1. System Architecture

The three main elements that compose the system are (i) a NXT mobile robot, (ii) an Android device for the remote control, and a pan IP camera. Since the experiment is carried out in a situation of teleoperation (i.e., indirect perception and action on the robot environment), a wireless connection is used to support the communication between the principal components of the architecture. Two different protocols of communication are applied. The Android-based remote control communicates with the NXT through Bluetooth technology. In addition, the connection between the IP camera and the smartphone is supported by Wi-Fi communication. The robot is connected to the IP camera thanks to a support library that permits the system integration between the two entities. Thus, the operators use the Android remote control device to interact with the whole system, which allows them to steer the mobile robot and receive a visual feedback from the pan IP camera. An Android application is developed and implemented on the smartphone to permit such an interaction. The tactile user interface enables the operator to control the trajectories of the vehicle, to choose the steering mode of the robot (manual vs. nonbiologic vs. biologic), to calibrate the pan camera, and to turn the system on or off.

### 2.2. Robot Behavior

**Figure 1:**
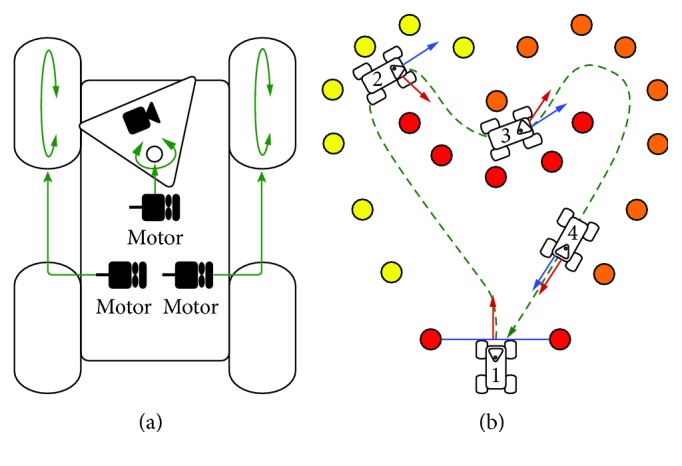
(a) Schematic representation of the robot (top view) designed for the study. Two independent motors drive the front wheels, and a third one controls the rotation of the pan camera. This mobile vision is implemented by default to promote a visual anticipation over the change of direction. (b) Illustration of the camera behavior in some specific locations of the path. The blue arrow indicates the instantaneous direction of the vehicle and the red arrow represents the orientation of the pan camera at the same moment. It is notable that the angle between the two arrows is inversely proportional to the radius of curvature of the robot trajectory. The more curved is the shape of the path (e.g., position 2), the larger is the angle between the orientation of the camera and the direction of the vehicle, and vice versa (e.g., position 4).

The vehicle is built on four wheels, employing a front-wheel-drive system (Figure 1(a)). The two front wheels are moved by two independent motors. The differential of speed between the right and the left wheel rotation allows the vehicle to turn. The pan camera is set on a mobile structure, which is moved by another motor. The orientation of the camera is determined automatically based on the direction of the robot, that is, the camera points toward the inside of the vehicle trajectory. Since any change of direction is systematically anticipated by a rotation of the camera proportional to the curvature of the vehicle trajectory, a visual prediction over the robot motion is provided to the operator. This mechanism inspired from the human behavior [12, 14] is implemented by default, because it facilitates the teleoperation [8, 10].  Figure 1(b) shows examples of this visuolocomotor coupling between camera and robot for different curves of the path.

## 3. Experimental Conditions

### 3.1. Manual Condition

**Figure 2:**
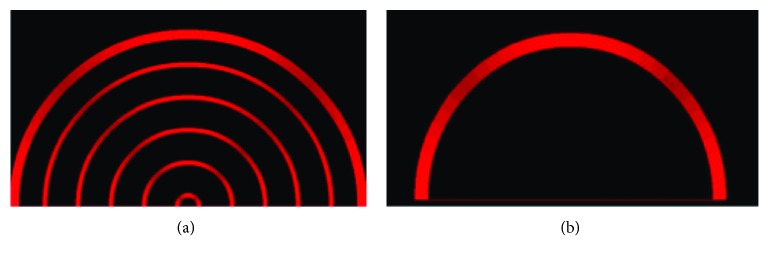
(a) Representation of the GUI for the manual mode of driving. Each concentric circle represents a different speed (the larger the radius of the semicircle, the higher the velocity). (b) User interface for the biological and nonbiological conditions. A single semicircle enables the user to directly control the direction of the robot and indirectly set the speed of the vehicle.

Both speed and direction of the vehicle are manually controlled by the operator in this experimental condition. Concentric semicircles that correspond to different speed levels are displayed on the control panel of the user's interface (Figure 2(a)). The bigger is the radius of the semicircle, the higher is the speed. Thus, the vehicle velocity is calculated based on the distance between the center of all concentric semicircles and the selected semicircle. The direction of the robot is determined by the angle between the vertical of the screen and the location of the user's fingertip. The range of angles goes from 0° to 180°, rotating counter clockwise. If the fingertip of the user is positioned between 0° and 90°, the robot turns right, with a curvature proportional to the angle between the vertical (90°) and the position of the finger (the more the location of the finger tends to 0°, the more the vehicle turns right). On the contrary, if the position of the finger is between 90° and 180°, the vehicle turns left (again, the radius of the curvature of the trajectory depends on the angle from the vertical of the screen). The controller of the robot is constantly waiting for an input sent from the graphic user interface, in order to update the direction and speed of the mobile platform.

### 3.2. Biological and Nonbiological Conditions

In these driving modes, the user has only to use the touchscreen interface to control the trajectory of the robot. The speed is automatically set according to the direction of the vehicle. In the biological condition, the 2/3 power law is used to calculate the speed, which is based on the instantaneous radius of the curvature of the robot trajectory. The maximum velocity of the robot is 30 cm/s, if the vehicle goes straight forward. In the case that the radius of curvature decreases (to the right or to the left), the robot's speed diminishes by a rate of one-third (see equation (1)). In the nonbiological condition, the velocity of the vehicle is also automatic, but it is not set according to the biological motion. The relationship between speed and geometry does not follow a power law, but a linear law described in

(2) $$\begin{array}{c} v_{t}=k\;r_{t}. \end{array}$$

**Figure 3:**
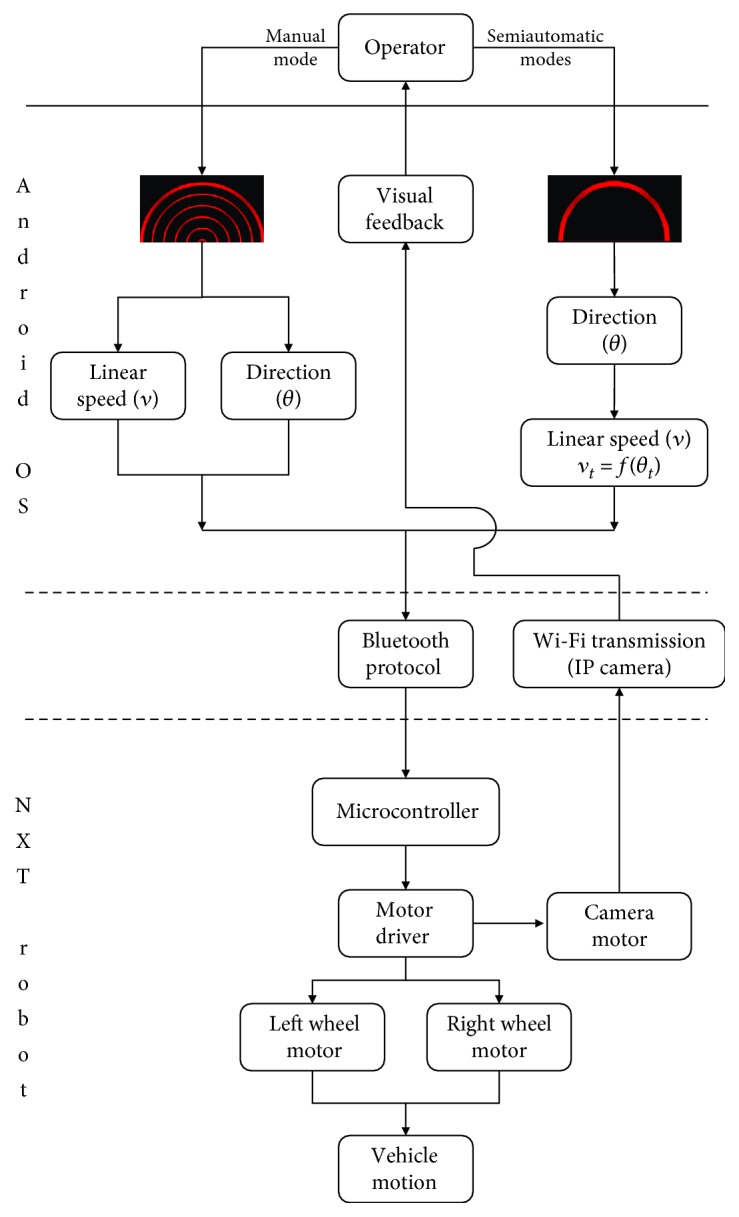
Block diagram of the two modes of remote control. If the operator picks the manual mode (left side), the speed and direction are controlled independently. On the contrary, if a semiautomatic mode is selected (right side), the robot speed is automatically calculated from the power law (biological condition) or linear law (nonbiological condition) function of the direction defined by the user.

Since it is not necessary to modulate manually the velocity, the graphic user interface is represented only by a single semicircle (Figure 2(b)). The semicircle allows the operator to control the trajectory of the robot. From the user's perspective, the way to steer the vehicle is identical to the manual mode of driving. The user has to interact with the left and right portion of the semicircle to turn left and right, respectively. The more the fingertip is located to the extremities of the semicircle, the more the robot turns sharply. The only difference between these two semiautomatic modes and the manual one is the fact that the velocity is indirectly and automatically set when the user chooses a determined direction. Precisely, the robot speed is proportional to the selected steering angle. Thus, if the power law is adapted to the remote control of an artefact, the matching between speed and steering angle should perfectly fit to the human's skills. On the contrary, because of its unnatural behavior, the nonbiological semiautonomous control should be more challenging for the teleoperator.  Figure 3 summarizes, through a block diagram, the differences between the manual and semiautomatic modes of control.

## 4. Experimental Protocol

**Figure 4:**
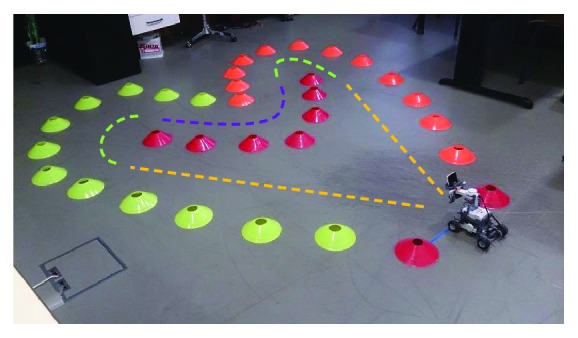
Picture of the experimental setting. The symmetric form of the path was chosen to easily alternate the course direction of the robot from one trial to the next: once clockwise and once counter clockwise. This alternation was designed to minimize the environment learning and a consequent machine-like driving of the vehicle. The two straight lines, two 150° bends, and one 90° bend are identified by broken yellow, green, and magenta lines, respectively. Note that these colors are added for a better understanding of the setup but were not visible during the experiment.

Thirty people (15 males and 15 females; 23.5 ± 3.5 years) took part in the experiment. All the participants had a normal or corrected-to-normal vision. The procedure conformed to the Declaration of Helsinki and was approved by the Ethical Review Board of the Nova University of Lisbon. The experiment was carried out in a classroom, where the subjects had to teleoperate the NXT vehicle through the Android-based mobile device. The instructions provided to the participants were to steer the robot as safe (the least collisions) and fast (the minimum completion time) as possible through a path delimited by plastic blocks. The entire distance of the route was approximately seven meters and consisted of numerous bends and changes in direction (curves and countercurves). The sequence of the course was as follows: (i) a straight line, (ii) an approximately 150° bend, (iii) a 90° reverse curve, (iv) another 150° bend, and (v) a final straight line (Figure 4). A blue adhesive strip marked the starting and finishing line. The symmetric shape of the setup was especially designed to carry out the route in both directions, clockwise and anticlockwise.

After a training session, all the subjects had to execute the trial twelve times: four repetitions in the manual mode, four repetitions in the biological mode, and four repetitions in the nonbiological mode. The order of the experimental conditions was counterbalanced from one subject to another so that ten individuals started with the manual control, ten others started with the biological control, and the last ten started with the nonbiological control. This counterbalancing was implemented to prevent a possible learning effect, which would bias the outcome of the study. For each of the principal conditions (manual vs. biologic vs. nonbiologic), the trial was performed twice clockwise and twice anticlockwise. The completion time, the number of collisions, and the robot trajectory were recorded at the end of each trial.

## 5. Results

The experimental data are statistically analyzed through ANOVA tests for multivariable comparisons and *t*-tests for the pairwise comparisons.

### 5.1. Completion Time

We first analyzed the time performance of the participants to complete the task. Results indicate that the completion time is significantly affected by the experimental sessions (*p* < .05). A pairwise analysis shows a significant difference between session 1 and session 4 (*p* < .03). This outcome indicates that the required time to guide the vehicle from the starting line to the finishing line diminishes significantly from session 1 to session 4. No interaction effects are detected between the sessions (1, 2, 3, and 4) and the main conditions (manual, biologic, and nonbiologic).

**Figure 5:**
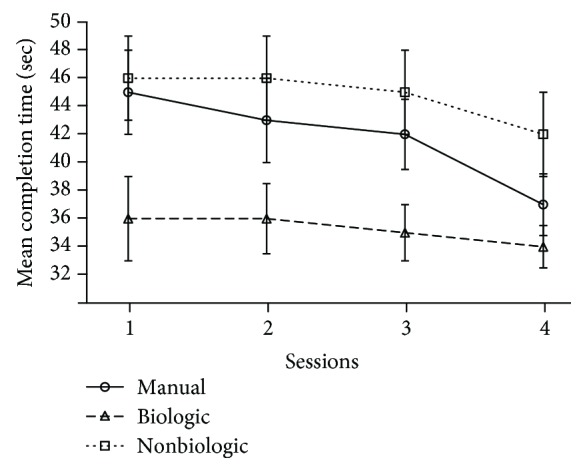
Representation of the mean completion time (in seconds) for each of the main conditions (manual vs. biologic vs. nonbiologic) against the four experimental sessions.

In addition, the comparison of the completion time between the three conditions indicates a significant difference (*p* < .005). As shown in Figure 5, the participants complete the task faster in the biological mode than in the manual (*p* < .01) and nonbiological (*p* < .005) steering modes. The pairwise analyses confirm the significant difference in session 1 (*p* < .01), session 2 (*p* < .05), and session 3 (*p* < .04). Nevertheless, this statistical difference vanishes in session 4, although the manual and nonbiological modes tend to remain slower than the biological. The reduction of the completion time over the sessions can be explained by a learning effect that occurs in all the conditions.

### 5.2. Number of Collisions

The assessment of the rate of collisions was also performed to complement the analysis. The statistical results indicate that the mean number of collisions is significantly different over the sessions (*p* < .03). The pairwise analysis shows a significant diminution of the collisions from session 1 to session 4 (*p* < .02). These outcomes point out that the subjects have improved the quality of their driving skills over the experiment. There is no effect of interaction between the four sessions and the main experimental conditions (manual, biologic, and nonbiologic).

**Figure 6:**
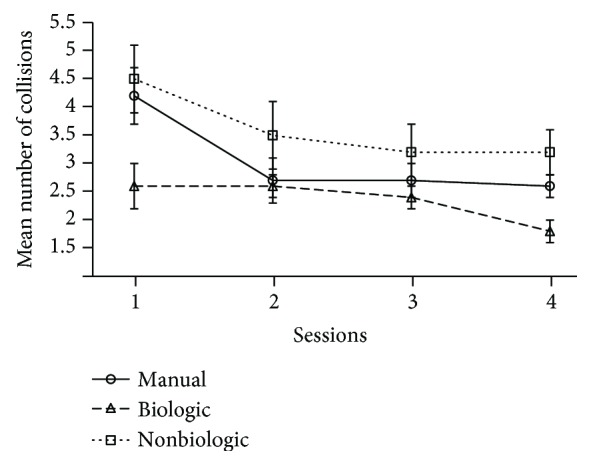
Representation of the average number of collisions for each of the main conditions (manual vs. biologic vs. nonbiologic) against the four experimental sessions.

The principal comparison between three conditions shows a significant difference over the whole sessions (*p* < .02). As plotted in Figure 6, more collisions occur in the manual and nonbiological conditions than in the biological condition. The statistical analysis session by session indicates a significant difference in session 1 (*p* < .01) and session 4 (*p* < .03). This last fact suggests that the learning effect does not enable the users in the manual and nonbiological modes to get steering skills as good as in the biological condition.

### 5.3. Trajectory Smoothness

The last results address the question of the movement kinematics through the analysis of the jerk in the control of the robot trajectory. One way to quantify the path smoothness is to calculate the instantaneous radius of curvature of each trajectory, then to evaluate the distribution frequency of the radius for all trials [40]. More specifically, the curve radius (*r*) is computed from the instantaneous linear velocity (*v*) divided by the instantaneous rotation speed (*w*), as described in

(3) $$\begin{array}{c} r_{\left(m\right)}=\frac{v_{\left(m/s\right)}}{w_{\left(radians/s\right)}}. \end{array}$$

Subsequently, the radius of curvature is converted into a decimal logarithm. Therefore, if the vehicle has a low linear speed and a high velocity of rotation, the curve radius is very small (<2), and gets smaller as the velocity of rotation increases. The result is a logarithmic value of *r* that is around zero. Conversely, if the vehicle combines a translation and a rotation (curvilinear trajectory), the curve radius is high (≥2) and its logarithm becomes superior to zero. A steering control in which the subject stops and turns in place provides a bimodal distribution of the curve radii, with one peak centered on null values of the logarithm and another peak centered on positive values. On the contrary, a curvilinear (or smooth) trajectory is characterized by a unimodal pattern of distribution centered on a value of the logarithm of the radius of curvature higher than zero. For each trajectory, the distribution of the logarithm of the curve radii is computed and distributed in three categories (small radii, curvilinear trajectories, and straight lines), according to a continuous scale of ranges that permits performing a statistical analysis of the results. To finish, we normalized the distributions, in which the occurrences of radii of curvature in each category are represented by a percentage of all the occurrences for each trajectory.

**Figure 7:**
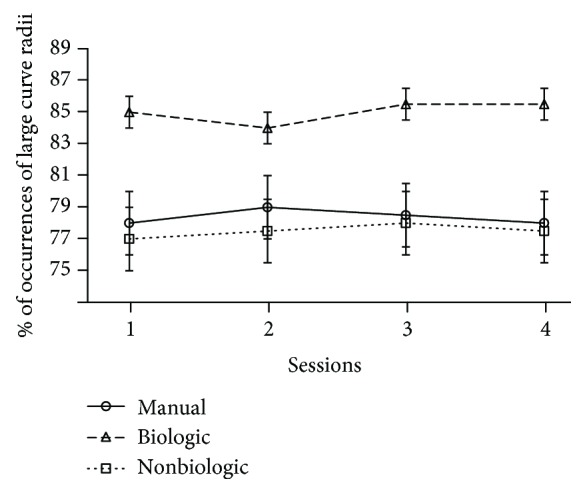
Representation of the average rate of large curve radii for each of the main conditions (manual vs. biologic vs. nonbiologic) against the four experimental sessions.

**Figure 8:**
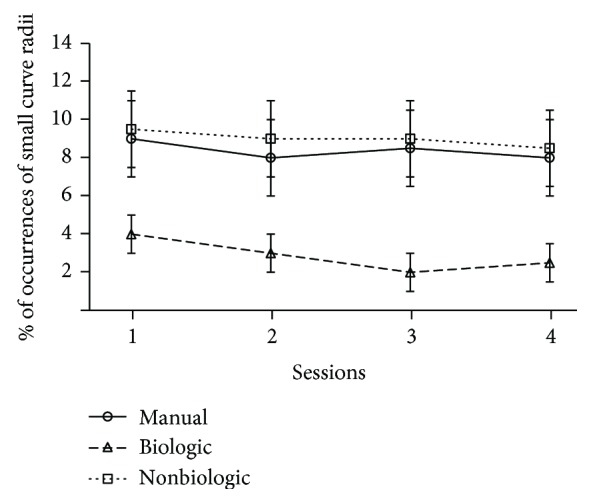
Representation of the average rate of small curve radii for each of the main conditions (manual vs. biologic vs. nonbiologic) against the four experimental sessions.

**Figure 9:**
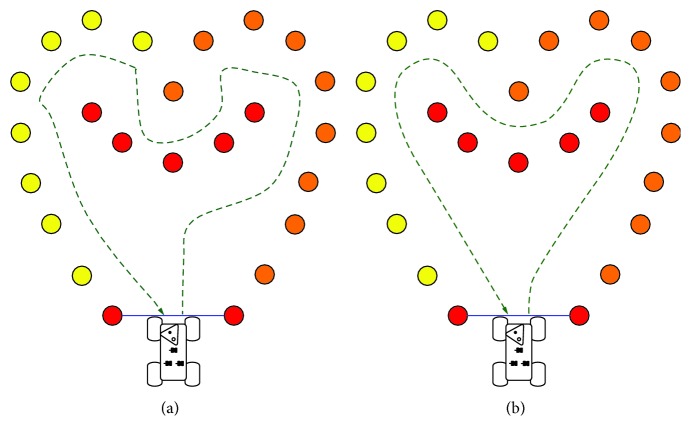
Typical example of a path (green dotted line) performed by a robot controlled in the manual or nonbiological mode (a). Notable is the sharp pattern that occurs before the main changes of direction. Sample of a path performed by a robot controlled in the biological mode (b). This condition is characterized by uniformly smoothed trajectories of the vehicle.

The distribution of large (Figure 7) and small (Figure 8) radii of curvatures is not the same whether the subjects interact with a robot that implements the human-like behavior or a robot that implements the two other modes of control. Thus, the percentage of occurrences of curvilinear trajectories is significantly higher in the biologic than in the manual and nonbiologic conditions (*p* < .01). Similarly, small radii and turn in place are statistically more frequent in the manual and nonbiological than in the biological condition (*p* < .01). In addition, these significant differences are maintained stable over the whole duration of the experiment. It means that four sessions are not enough to provide the teleoperator with a learning effect that could counterbalance the benefit of the bioinspired semiautonomous mode, in terms of the rate of both jerky trajectories (*p* < .01, at session 4) and smooth movements (*p* < .01, at session 4). The difference of steering control can be confirmed by the visualization of the typical paths recorded for each experimental condition (Figure 9). This advanced analysis of the motor performance shows that the operator tends to maximize the smoothness of the robot trajectories, when the vehicle replicates the natural human scheme described by the power law.

## 6. Discussion

This study consisted in analyzing the effect of the implementation of the 2/3 power law on the steering control of a vehicle. Three experimental conditions were compared. In the first condition, the participant had to manually control both the speed and the direction of the robot. In the second condition, the velocity of the vehicle was automatically set according to the bioinspired model. Lastly, in the third condition, which was used as a control, the robot speed was automatically calculated through an equation that violated the biological motion. The task of the subjects was to remote control the robot, in order to complete the course as safe and fast as possible. The performance of the participants was recorded on four sessions. The statistical analyses indicate that the number of collisions and the completion time diminish significantly over the sessions. This overall improvement of the performance seems to be related to a learning effect. The main comparison of the study shows that the precision and velocity to accomplish the task are significantly better in the biological condition than in the manual and nonbiologic conditions. Since the speed control is automatic in the biological condition, less sensorimotor resources and mental workload of the teleoperators are required to complete the task. This aspect brings an advantage for the individuals, who can focus their attention on the guidance of the vehicle. Nevertheless, the fact that the nonbiological condition is significantly worse than the biological condition means that the automatic setting of the speed must replicate certain characteristics of the natural movement to be effective.

The comparison of the raw performances (speeds and collisions) was complemented by a more advanced assessment based on the analysis of the robot's kinematics. The radii of curvature of the vehicle trajectory were analyzed, in order to evaluate the smoothness of the movements. Like the raw performance, this parameter shows the benefit of implementing a human-like behavior in the robot's way of working. The trajectories are significantly smoother when the power law is integrated into the robot than when this bioinspired model is absent. Remarkably, the study shows that the advantage of the biological law lasts until the end of the experiment, which supposes a stronger impact of the implementation than the learning effect. This result suggests that the power law and minimum jerk are indeed related to each other. Such an outcome is supported by studies that tend to demonstrate that the 2/3 power law is an optimal solution to smooth the trajectory, because it sets the normal component of the jerk to zero [32, 39, 41]. In addition, it seems that this law satisfies the principle of least action, which states that the amount of work required to complete a trajectory is minimal if the movement obeys the 2/3 power law [42]. This observation is consistent with an experiment of telemanipulation showing that the motor skill and performance is negatively correlated with the mental workload of the surgeon during robot-assisted surgery [43]. This finding suggests that the smoothness of the robot movements controlled by an operator could be used as an indirect measurement of the workload.

Furthermore, the fact that the control of a nonanthropomorphic robot is significantly improved when the artefact behaves according to the 2/3 power law supports the hypothesis of the CNS (Central Nervous System) origin of this law [21, 44]. Viviani and Flash [32] described a correlation between the power law and movement prediction, in order to plan and choose the best trajectory. More precisely, these authors underline that the estimation of the trajectory geometry must be accessible to the motor control system as a part of the internal representation of the predicted movement intention. This is a fundamental feature of the locomotion that requires to program changes in direction one step ahead, in order to overcome the delays due to biomechanical inertia [45]. This motor coordination seems also to occur during the execution of a movement mediated by an artefact, which suggests that this control rule is characteristic of a general scheme of the organization of the action. This observation is supported by the replication of the two-thirds power law in a mobile robot with quite different (bio)mechanics than the human being, which would confirm the hypothesis that this law is not dependent on peripheral biomechanic factors [30, 46], but as issued from an internal model of the movement planning [21, 47].

Moreover, the fact that the operator observes a mobile device that has human-like kinematics can also explain the advantage of the biological mode over the nonbiological. Several experiments show that the observation of a biologically plausible movement facilitates the simultaneous execution, by the observer, of a congruent action [2, 48]. Mirror neurons, and more specifically the Action Observation Network (AON), seem to be involved in this process [49]. In fact, several neuroimaging studies have shown that the activation of the mirror neuron system areas is modulated by the observer's motor experience [50, 51]. According to predictive coding, the optimal state is a minimal prediction error at all levels of the AON, which is achieved when the observed actions match predicted actions (based on prior visuomotor experience) as closely as possible [52, 53].

To conclude, it is important to mention that it is not always an advantage to automate some parameters of the artefact in a situation of human-machine interaction. Our study suggests that the characteristics of the human being must be taken into account to create appropriate usability rules. Here, the proposed method is to implement a bioinspired behavior to automate the velocity of a robot. In the case study of the teleoperation of a mobile device or robotic arms, the anthropocentric approach seems to be efficient. A current trend in the automobile industry is to produce more and more autonomic vehicles [54], which is in a certain sense in contradiction with the will of the drivers, who want to keep the control on the technology. Our results suggest that modeling and implementing human-like behaviors in the machine, such as the two-thirds power law [23] or Fitts' law [55], is a promising alternative approach for the automatization of key processes in the artefact's way of working. The advantage of such a method comes from the fact that a car behaving as a living being can be easily understood and appropriated by the end user [36]. Future work will consist in exploring other approaches based on machine learning or reinforcement learning to train the robot to acquire human-like behaviors and, also, improving the transparency of the remote control by providing the operator with natural user interfaces, such as the Kinect, to interact with the machine [17].

## Data Availability

The data used to support the findings of this study are available from the corresponding author upon request.
